# Can new immunoassay techniques improve bladder cancer diagnostics With protein biomarkers?

**DOI:** 10.3389/fmolb.2020.620687

**Published:** 2021-02-15

**Authors:** Yuri M. Shlyapnikov, Ekaterina A. Malakhova, Andrey Z. Vinarov, Andrey A. Zamyatnin, Elena A. Shlyapnikova

**Affiliations:** ^1^ Institute of Theoretical and Experimental Biophysics RAS, Pushchino, Russia; ^2^ Institute for Urology and Reproductive Health, Sechenov First Moscow State Medical University, Moscow, Russia; ^3^ Belozersky Institute of Physico-Chemical Biology, Lomonosov Moscow State University, Moscow, Russia; ^4^ Institute of Molecular Medicine, Sechenov First Moscow State Medical University, Moscow, Russia; ^5^ Department of Biotechnology, Sirius University of Science and Technology, Sochi, Russia

**Keywords:** bladder cancer, protein biomarkers, immunoassay, magnetic labels, microarray

## Abstract

The search for new diagnostic tests for cancer or ways to improve existing tests is primarily driven by the desire to identify the disease as early as possible. In this report, we summarize the current knowledge of the most promising diagnostic protein bladder cancer (BC) markers reported over the last decade. Unfortunately, analysis of published data suggests that a reliable, highly sensitive biomarker test-system based on ELISA for detecting BC has not yet been developed. The use of more sensitive assays to detect ultra-low concentrations of biomarkers not available for ELISA, could be very beneficial. Based on the literature and pilot experimental data, we conclude that a highly sensitive immunoassay using microarrays and magnetic labels, could be an effective and cheap technique suitable for the detection of diagnostically relevant BC biomarkers.

## Introduction

When cancer is detected at early stages, tumor removal or other kinds of treatment can lead to long-term survival. However, diagnostics often require highly invasive procedures such as biopsy, whereas non-invasive approaches are still a significant challenge. Measuring serum biomarkers is considered to be a promising method for non-invasive cancer diagnosis. From this point of view, urinary cancers stand out among various forms of cancer, since not only serum, but also urine can be a source of biomarkers. The search for biomarkers of urinary tract cancer has been actively pursued in recent decades, but so far none of them has found application in clinical practice (Babjuk et al., 2019). This primarily refers to bladder cancer, which is the subject of the present Perspective. This is probably due to the fact that new biomarkers demonstrating high efficiency in research laboratories are not sufficiently reliable when used for screening a large number of patients. The relevance of the topic is confirmed by a large number of articles and reviews published in recent years, for example (Maas et al., 2019; Ng et al., 2020; Chakraborty et al., 2019; Kim et al., 2020; Batista et al., 2020; Faiena et al., 2019; Matuszczak and Salagierki, 2020; Miyamoto et al., 2020; Carando et al., 2021). The authors are constantly systematizing and analyzing new data to select biomarkers for both diagnosing BC and monitoring treatment response. Various substances can act as biomarkers of bladder cancer: proteins, metabolites, specific RNA or DNA sequences, and others (Supplementary Table S1). This report is focusing only on protein diagnostic urine biomarkers and the sensitivity of the methods used to identify them. Based on the literature and pilot experimental data, we hypothesize that the use of more sensitive assays that detect ultra-low concentrations of biomarkers unavailable for ELISA, are promising for diagnosing bladder cancer.

### Conventional immunoassays in BC diagnostics

Specificity and sensitivity are the two main factors determining the effectiveness of a diagnostic test. Table 1 provides information on the most promising protein BC biomarkers, both single and panel, with the best values of these parameters. We do not report data on well-documented FDA-approved urine tests for BC (Lopez-Beltran et al., 2019; Santoni et al., 2018; Wang et al., 2017; Glas et al., 2003) due to their low sensitivity, as already noted. As follows from Table 1, some biomarkers demonstrate both high sensitivity and specificity exceeding 90%.

**TABLE 1:** Potential diagnostic protein urine biomarkers for detecting bladder cancer.

Biomarker	Method of the detection	Sensitivity, %	Specificity, %	Accuracy, % or AUC	Notes	References
BLCA-4	Meta-analysis of 9 studies	93	97	AUC=0.961	Increased expression in the early stages	Cai et al. (2015)
	ELISA	97.7	100	–	Cutoff of A =13 units/μg protein	Konety et al. (2000)
Hyaluronic acid (HA)	ELISA	83.1	90.1	86.5	High-grade tumor detection	Lokeshwar et al. (2000)
Hyaluronidase (HAse)	Meta-analysis of 8 studies	83,4	86	AUC=0.91	PPV=89%	Liang et al. (2017)
	Zymography	89.4	89.4	AUC=0.948	–	Eissa et al. (2015)
Cytokeratin fragm. 8 and 18	ELISA (UBC® Rapid Test)	86.9	90.9	AUC=0.75	High-grade tumor detection Cut-off=10 ng/ml	Southgate et al. (1999); Ecke et al. (2017)
Cytokeratin-19	Meta-analysis of 8 studies	82	80	AUC=0.87	–	Huang et al. (2015)
Survivin	ELISA	35	98	–	Cut-off = 33 pg/ml High-grade tumor detection	Gleichenhagen et al. (2018)
Survivin+ Cytokeratin fragm. 8 and 18	ELISA+UBC® in combination	82	95	AUC=0.91	–	
Apo-A1	ELISA	91.6	85.7	AUC=0.928	Control/BC=18/30 ng/ml	Li et al. (2011)
ORM1	ELISA	91.96	94.34	93 AUC=0.965	–	Li et al. (2016)
sFas	ELISA	88.03	91.9	AUC=0.912	Cut-off=174 pg/ml	Srivastova et al. (2014)
HtrA1	ELISA	92.65	95.59	94	PPV = 95.45 NPV = 92.86	Lorenzi et al. (2013)
	Western-blot	–	–	AUC=0.983	–	
Tumor M2-PK	ELISA	82	–	–	–	Liu et al. (2019)
CD147	ELISA	97	100	–	–	Bhagirath et al. (2012)
HA; HAse	ELISA	91.2	84.4	88.3	–	Lokeshwar et al. (2000)
	Meta-analysis of 8 studies	90.8	82.5	AUC=0.94	–	Liang et al. (2017)
CCL18	ELISA	86	87	AUC=0.919	–	Urquidi et al. (2012)
CCL18; PAI; CD44		–	–	AUC=0.938	–	
APOA1; APOA2; APOB; APOC2; APOC3	BioPlex assay or Western-blot	–	–	–	Early detection of BC	Chen et al. (2013)
SAA4+ProEGF		–	–	AUC=0.8	–	
Coronin-1A; A4;g-Synuclein; Semenogelin-2; DJ-1	ELISA (non-invasive BC)	79.2	100	85.3 AUC=0.92	Perfect concordance between WB and RT-PCR data	Kumar et al. (2015)
	Western-blot (non-invasive BC)	93.9	96.7	94.8 AUC=0.98		
	ELISA (invasive BC)	86.4	100	90.6 AUC=0.94		
	Western-blot (invasive BC)	100	100	100		
ANG; APOE; CA9; Il8; MMP9; MMP10; PAI1; VEGF	Non-invasive BC ELISA	92	97	–	–	Goodison et al. (2012)
ANG; APOE; A1AT; CA9; IL8; MMP9; MMP10; PAI1; SDC1; VEGF	Multiplex bead-based immunoassay, Oncuria^TM^	–	–	–	Minimal detected dose (MDD): from 0.295 pg/ml in IL8 to 31.1 pg/ml in APOE	Furuya et al. (2020)

ECL, electrochemiluminescent.

The question arises as to why these biomarkers are not used to diagnose bladder cancer. Unfortunately, the available data are generally inconclusive. No comparative studies with a large enough sample size have been conducted to test these biomarkers and convince clinicians of their reliability (Babjuk et al., 2019). Although many studies to date have focused on systematic reviews and meta-analysis to assess the accuracy of various biomarkers (for example, Masuda et al., 2018; Chou et al., 2015; Kojima et al., 2017), the meta-analysis itself has certain limitations (Huang et al., 2015). In addition to the authors' use of different threshold values in specific investigations, the choice of studies for meta-analysis was not exhaustive, and incomplete data processing could lead to distortion of the final result.

Since the known biomarkers detected with conventional immunoassay methods are not used by clinicians, new tools are needed for early BC diagnosis. Unfortunately, over the past few years, there has been very little information on the discovery of novel protein tumor markers.

Currently, attempts are under way to use the marker of urinary tract infection, urinary lactoferrin, for diagnosis and prognostication of BC (Matsumura et al., 2020). An ongoing study is evaluating the potential of PD-L1 as a prognostic biomarker that correlates with the pathological stage of BC (Gulinac et al., 2020). Efforts to improve the efficiency of the assay have mainly focused on pooling known biomarkers. The eight-biomarker panel was found to achieve a sensitivity of 92% and a specificity of 97% (Goodison et al., 2012). The inclusion of PAI-1 and CD44 in a panel for detecting CCL18 was shown to make the test more reliable (Urquidi et al., 2012). Chen et al. (2013) reported that the proteins of serum amyloid A in combination, might be useful in the early detection of bladder tumors. Thus, the first report on the use of a combination of SAA4 and ProEGF as a new ВС biomarker appeared. Liang et al. (2017) showed the diagnostic accuracy of the combination of hyaluronic acid and hyaluronidase. Kumar et al. (2015) found all five biomarkers to be required for accurate assay. Gleichenhagen et al. (2018) evaluated the performance ELISA for survivin, UBC® test measuring cytokeratin fragments 8 and 18, and the combination of both assays. They confirmed the benefit of using marker panels. Using Luminex xMAP technology, Furuya et al. (2020) developed the first multiplex bead-based immunoassay; minimal detected dose of 10 urinary biomarkers ranged from 0.295 pg/ml in IL8 to 31.1 pg/ml in APOE.

Thus, a combination of several biomarkers, each of which carries independent diagnostic information, provides greater sensitivity and accuracy for BC detection. However, unlike urine-based multiplex RNA analysis (Darling et al., 2017), no multiplex protein assay is on the horizon for clinical use.

Since traditional methods yield moderate results, fundamentally new ways are required. We propose a new approach focused on increasing the sensitivity of analytical techniques used to detect protein biomarkers. The most common techniques for detecting protein biomarkers are ELISA and the Western blot (WB). These well-developed and widely used methods, like any other analytical technique, have a certain limit of detection (LOD). This limit is governed by fundamental factors such as diffusion restrictions during delivery of the analyte to surface probes and the thermodynamics of the antigen-antibody complex (Shlyapnikov et al., 2020). If the concentration of the biomarker is below this limit (the typical value of which in the case of ELISA is about 10–100 pg/ml), the presence of the biomarker will be missed. The central question of the present Perspective is: what if there are cancer biomarkers with concentrations below the LOD of ELISA? Then the use of conventional immunoassays excludes any possibility of their detection. To reveal their diagnostic potential, more sensitive immunoassay methods with an LOD lower than that of traditional methods are required. Many such techniques have been described, as shown below. Importantly, we do not claim that lowering the LOD of immunoassay could improve the detection of known biomarkers. Even the conventional immunoassays with a relatively high LOD often give false positive results, since cancer biomarkers are present not only in patient samples, but also in healthy subjects. To remove false positives, the threshold concentration of a biomarker should be chosen. Thus, we see no reason to use a more sensitive assay to detect known biomarkers.

### Ultrasensitive immunoassay for detecting urinary cancer-retina antigens in BC patients

Many different approaches for increasing immunoassay sensitivity have been proposed. These include well-known methods such as immune-PCR or tyramide-amplified ELISA (Gong et al., 2012) for which commercial ultrasensitive kits have been developed, and modern sensing techniques, such as electrochemical detection employing polymeric enzyme nanoparticles as labels (Dhanapala et al., 2020). However, if a signal determined by specific analyte-antibody interactions is amplified, all concomitant non-specific interactions are also enhanced. When real biological samples of complex composition are analyzed, the challenge is to sense small amounts of analyte in the presence of a huge excess of various interfering substances (Morozov et al., 2007). This may require not only a low LOD, but also a very high assay specificity, which is unreachable for many amplification-based techniques. Amplification-free, highly sensitive immunoassays, based mainly on fluorescence, have also been reported. However, to detect several thousand molecules, they require high-cost sophisticated optical hardware.

The “active” bead-linked immunoassay technique stands apart from all of the above-mentioned methods and provides a unique combination of sensitivity and specificity (Shlyapnikov et al., 2020; Dhanapala et al., 2020; Morozov and Morozova, 2006). As in the case of traditional sandwich immunoassay, analyte molecules are captured with antibodies on a microarray fabricated on a special low-adhesive substrate (Shlyapnikov et al., 2014). The microarray is then installed in a flow cell, under which a magnet is located. Its surface is then scanned with micrometer-sized magnetic beads coated with detecting antibodies in a laminar flow. The beads are retained in the microarray active zones due to specific antigen-antibody interactions and are detected using a dark-field optical microscope. A single intermolecular bond is able to tether a bead, and thus individual molecules can be marked. This defines an extremely low LOD that can reach zeptomolar values.

The principal unique feature of this method is that specific and non-specific interactions are differentiated based on mechanical force rather than an equilibrium constant. The force acting on a bead is controlled by the shear rate, which is an additional degree of freedom to optimize the analysis. Force discrimination allows a unique assay specificity to be achieved; it makes it possible to distinguish between cross-reactive antigens that are thermodynamically equivalent (Morozova and Morozov, 2008) and to detect analytes in the presence of a 10^11^ molar excess of other proteins (Shlyapnikov et al., 2020). At the same time, this detection technique is fast and usually takes only one to two minutes. It also allows multiple biomarkers to be detected simultaneously using multicomponent antibody microarrays. Finally, the main advantage of the bead-linked immunoassay is its technical simplicity and low cost. The flow cell and its periphery do not contain any sophisticated hardware (Morozov and Morozova, 2006). Moreover, the consumption of antibodies, which can significantly affect the analysis cost, is extremely low (Shlyapnikov et al., 2020), making the total cost comparable or even lower than that of ELISA or WB. Thus, we believe this approach to be very effective for the ultrasensitive simultaneous detection of various protein tumor markers that cannot be efficiently detected with common immunoassays.

Proteins specific to immune privileged zones, such as cancer-retina antigens, were shown to be aberrantly expressed in malignant tumors (Golovastova et al., 2014). Among these proteins, recoverin (Golovastova et al., 2016) and arrestin (Baldin et al., 2019; Kallifatidis et al., 2019) were found to be highly predictive biomarkers of renal cell carcinoma and BC. However, they have never been considered as low-invasive blood or urine biomarkers. To support our idea, we applied a novel bead-linked immunoassay to detect trace amounts of these proteins in urine samples from bladder cancer patients and healthy controls. We also compared its performance with a conventional immunoassay technique such as ELISA. The relevant experimental details are given in Supporting Information. The resulting LOD was 0.1 pg/ml for both analytes. Calibration curves for arrestin and recoverin are shown in Supplementary Figure S1. The results of quantitative determination of arrestin and recoverin in urine samples from BC patients and healthy controls obtained using magnetic beads detection, are presented in Table 2.

**TABLE 2:** Arrestin and recoverin concentrations in urine of BC patients and controls measured using an ultrasensitive microarray-based immunoassay.

BC patients			Healthy controls		
#	Arrestin, pg/ml	Recoverin, pg/ml	#	Arrestin, pg/ml	Recoverin, pg/ml
1	< 0.1	8 ± 5	1	0.8 ± 0.5	<0.1
2	< 0.1	8 ± 5	2	< 0.1	<0.1
3	< 0.1	<0.1	3	< 0.1	0.5 ± 0.3
4	> 10	<0.1	4	< 0.1	<0.1
5	< 0.1	5 ± 2	5	< 0.1	<0.1
6	3 ± 1	<0.1	6	< 0.1	<0.1
7	<0.1	7 ± 4	7	< 0.1	0.6 ± 0.4
8	<0.1	9 ± 6	8	< 0.1	<0.1

**FIGURE 1:**
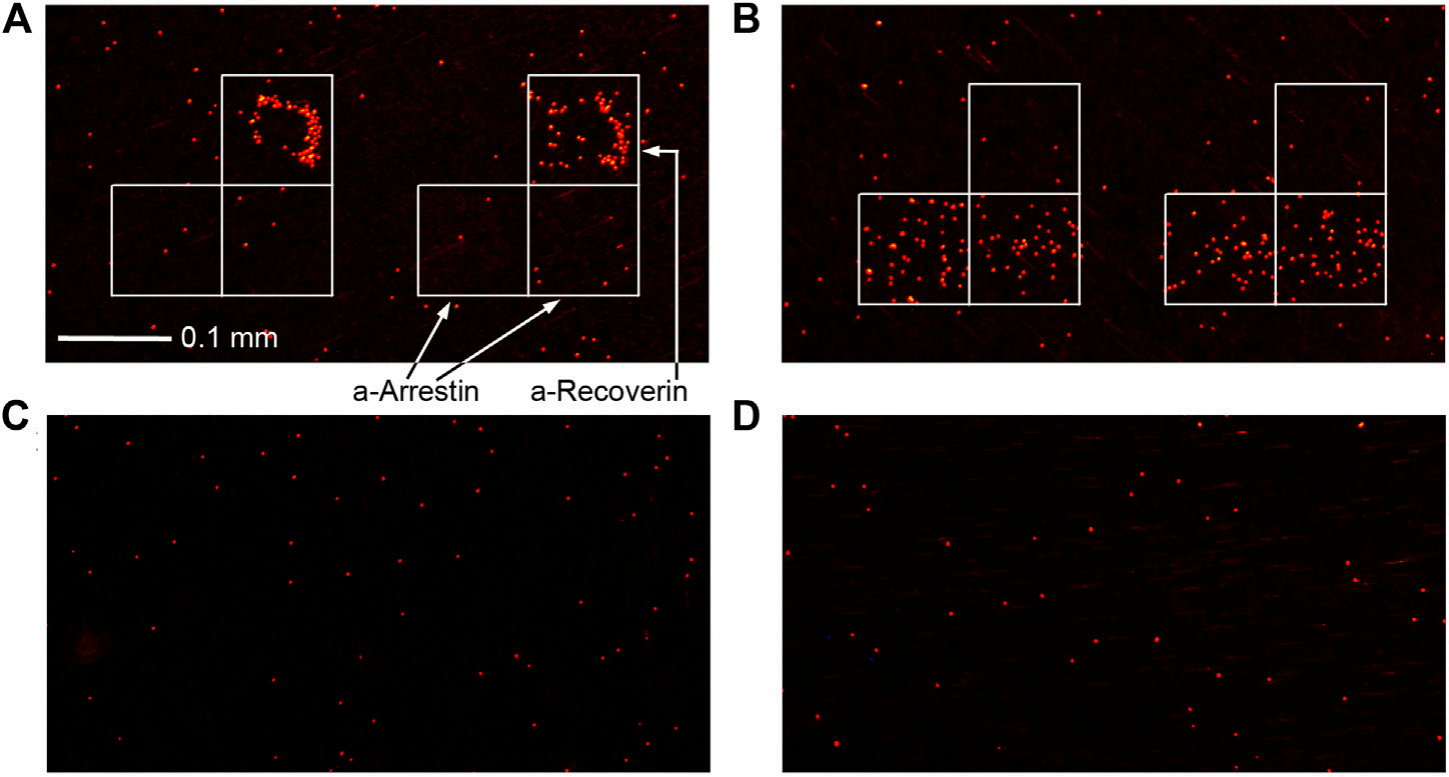
Immunoassay results of urine from BC patients **(A)** #1 and **(B)** #6 and healthy volunteers **(C)** #4 and **(D)** #5 (Table 2).

The sensitivity of arrestin or recoverin alone was 25% and 63%, respectively, while the sensitivity of their combination when the result was considered positive if either of the two analytes was detected, was 88%. Although three out of eight controls were also positive for any of the antigens, their detectable concentration was significantly lower than in samples from BC patients. Hence, a threshold value can be chosen to effectively discriminate between BC and control groups. For the reported data, a threshold of 1–2 pg/ml provides 88% sensitivity and 100% specificity within this small sample. Since their concentrations in patients were lower than the LOD of traditional ELISA, the latter is obviously ineffective for diagnosing BC with these tumor markers. This was confirmed directly by performing ELISA of the same samples. The LOD of both arrestin and recoverin was 1 ng/ml by ELISA (Supplementary Figure S2), and none of these analytes were detected in any of the samples by this common assay (Supplementary Table S2). Although only two protein markers were used in this study, the presented method can detect up to 20 biomarkers in one assay.

In this Perspective, we point out the serious issue of the lack of sufficiently effective protein biomarkers for the early diagnosis of certain forms of cancer, in particular, BC. We hypothesize that the use of common immunoassay techniques significantly limits the range of protein biomarkers, and many new biomarkers could be found using more sensitive methods that allow lower analyte concentrations to be detected. As a “proof-of-concept”, we demonstrate the detection of ultra-low concentrations of cancer-retina antigens in BC urine samples. Thus, we conclude that the use of immunoassays that have a lower limit of detection than conventional ones, could substantially advance cancer diagnostics with protein biomarkers.

## Data Availability

The raw data supporting the conclusions of this article will be made available by the authors, without undue reservation.
